# Comparison of visual outcomes in adult patients with different types of developmental cataracts after toric multifocal intraocular lenses implantation

**DOI:** 10.1007/s00417-023-06148-4

**Published:** 2023-06-29

**Authors:** Jiying Shen, Dongmei Ma, Lei Cai, Limei Zhang, Haike Guo, Jin Yang

**Affiliations:** 1 Department of Ophthalmology, Shanghai Heping Eye Hospital, Shanghai, China; 2 Department of Ophthalmology and the Eye Institute, Eye and Ear, Nose, and Throat Hospital, Fudan University, Shanghai, China; 3 The Key Laboratory of Myopia, Ministry of Health, Shanghai, China; 4 Shanghai Key Laboratory of Visual Impairment and Restoration, Shanghai, China; 5 Key National Health Committee of the Key Laboratory of Myopia, Fudan University, Shanghai, China; 6 The Key Laboratory of Myopia, Chinese Academy of Medical Sciences, Shanghai, China

**Keywords:** Developmental cataract, Astigmatism, Toric multifocal IOL, Visual quality, Cataract surgery

## Abstract

**Purpose:**

To analyze and compare the visual performance and patient satisfaction following the implantation of toric multifocal intraocular lenses (TMIOLs) in adult patients with different types of developmental cataracts (DC) accompanied by corneal astigmatism (CA).

**Methods:**

This is a prospective observational cohort study. Patients diagnosed with DC aged 18–30 years were divided into three groups according to the anatomic location of the lens opacity: cortical, nuclear, and posterior subcapsular (PSC) groups, and implanted with TMIOLs. Visual acuity (VA), postoperative refractive astigmatism (RA), intraocular lens (IOL) rotation, high-order aberrations (HOAs), modulation transfer function (MTF) curve, and Strehl ratio were compared. The functional vision and incidence of photic phenomena were surveyed using questionnaires.

**Results:**

Fifty-five eyes of 37 patients were enrolled and completed a 1-year follow-up. The mean CA was 2.06 ± 0.79 D preoperatively, and the mean RA was 0.29 ± 0.30 D 3-month postoperatively. The IOL rotation was 2.48° ± 1.89°, with no deviation > 10°. At 12 months, mean uncorrected distance VA improved from 0.93 ± 0.41 preoperatively to 0.08 ± 0.08 logarithm of the minimum angle of resolution (logMAR), mean uncorrected near VA increased from 0.45 ± 0.30 preoperatively to 0.12 ± 0.11 logMAR, and mean uncorrected intermediate VA was 0.14 ± 0.08 logMAR. The cortical and nuclear groups displayed better improvements in uncorrected near and intermediate VA than that in the PSC group. Similar results were observed in the 3-month defocus curves, HOAs, MTF curve, halo incidence, and near vision satisfaction.

**Conclusion:**

In adult patients with DC accompanied by CA, TMIOLs implantation achieved good postoperative visual outcomes and significantly reduced glasses dependency. Patients with cortical or nuclear lens opacity showed better whole-course VA and quality of vision, while patients with PSC opacity showed unsatisfactory near vision and suffered more photic phenomena.

**Supplementary Information:**

The online version contains supplementary material available at 10.1007/s00417-023-06148-4.


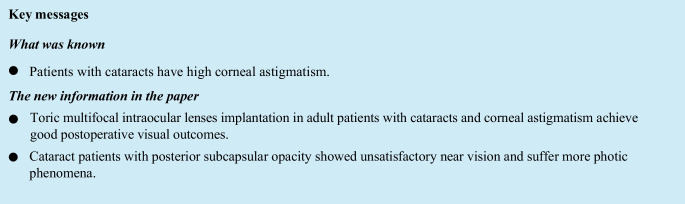


## Introduction

Developmental cataract (DC) is a clinically rare and indistinguishable disease. Unlike congenital cataract (CC), which is the leading cause of vision disabilities and treatable blindness in children [1], DC is not easy to be detected in the early stage and usually does not affect the visual function of patients in the childhood and teenage years because of its mild and static disease forms. Patients with DC usually consult ophthalmologists on experiencing significant vision loss between the ages of 18 and 30 years [2]. These working-age patients usually present with higher corneal astigmatism (CA) and have a higher demand for good eyesight and clear whole-course visual acuity (VA) [3–6]. Meeting these requirements has always been a source of concern for ophthalmologists.

Astigmatic correction improves visual outcomes such as reading performances and uncorrected VA [7, 8]. In recent years, toric monofocal intraocular lenses (IOLs) have achieved great success in CC and DC with CA. A previous study reported that the postoperative refractive astigmatism (RA) of 76 eyes in 51 children declined from 1.56 ± 2.13 D preoperatively to 0.55 ± 0.40 D after the implantation of toric monofocal IOLs, with 74% of patients having an uncorrected distant visual acuity (UDVA) of at least 20/40 [9]. A comparative study reported the mean postoperative refractive cylinder in the toric group was significantly lower than that in the non-toric group (0.50 ± 0.39 D vs. 2.05 ± 0.39 D); therefore, UDVA in the toric group was superior [10]. Additionally, using toric multifocal intraocular lenses (TMIOLs) in age-related cataracts (ARC) with CA provides better whole-course VA and quality of vision [11, 12]. However, to the best of our knowledge, there have been few reports on the visual outcomes and satisfaction after TMIOL implantation in adult patients of DC with CA. Therefore, we divided these patients into three groups according to the anatomic location of the lens opacity to investigate the feasibility and clinical benefits of TMIOLs (At Lisa 909 M) implantation in this population and compare the differences in visual satisfaction and quality of vision among the three groups.

## Methods

### Study design and participants

This prospective observational cohort study included adult patients diagnosed with DC with CA. The patients were hospitalized for cataract surgery and At Lisa 909 M IOLs implantation between March 2019 and January 2021 at the Shanghai Heping Eye Hospital, Shanghai, and the Eye and Ear, Nose, and Throat (ENT) Hospital of Fudan University, Shanghai. The inclusion criteria were as follows: (1) age 18–30 years, (2) diagnosis of DC, (3) surgically treated for the first time, (4) preoperative regular with-the-rule (WTR) CA > 1.25 D, against-the-rule (ATR) CA > 0.75 D, or oblique astigmatism > 1.0 D [13]. The exclusion criteria were: (1) amblyopia or previous best-corrected distance visual acuity (BCVA) < 20/25 in the teenage years; (2) diagnosis of age-related, complicated, traumatic, metabolic, or toxic cataracts; (3) posterior polar cataracts, which may have posterior capsular defect; (4) neural, retinal, and choroid diseases. The inclusion and exclusion criteria were applied according to the diagnosis, medical history, progress notes, and other medical records. The study was approved by the Institutional Review Board of the Eye and ENT Hospital of Fudan University and the Shanghai Heping Eye Hospital. All procedures adhered to the tenets of the Declaration of Helsinki, and written informed consent was obtained from the patients.

Considering that DC is similar to CC in morphology, DC is often categorized using the classification methods of CC [14–16]. In this study, the patients were divided into three groups according to the anatomic location of the lens opacity [6], the nuclear (nuclear and zonular cataracts), cortical (punctate and cerulean opacities), and posterior subcapsular (PSC) groups. All patients had a follow-up period of 1 year or more.

### Intraocular lens

At Lisa 909 M IOL (Carl Zeiss Meditec AG) is a single-piece, foldable, acrylic TMIOL made of hydrophilic acrylate with a hydrophobic surface. It has a four-haptic design, an overall diameter of 11.0 mm, and an optic diameter of 6.0 mm. It is independent of pupil size, and the power distribution between the distance and near foci is asymmetrical (65% for distance focus and 35% for near focus) [17, 18].

### Preoperative examination

UDVA at 5 m, BCVA at 5 m, and uncorrected near visual acuity (UNVA) at 40 cm were recorded in logarithm of the minimum angle of resolution (logMAR) units. A full ophthalmologic examination using slit-lamp biomicroscopy, fundoscopy, and intraocular pressure measurement was performed for each patient. Corneal topography (Pentacam, Oculus Optikgeraete GmbH; Wetzlar, Germany), optical biometry (IOL-Master 700, Carl Zeiss Meditec AG), higher-order aberrations (HOAs), and Strehl ratio (SR) (the HOYA iTrace ray-tracing system, Tracey Technologies, Houston, TX) were measured before the surgery.

### Surgical technique

The phacoemulsification surgery was performed by Dr. J. Y. using a standardized surgical technique under surface anesthesia. Two marks of 0° and 180° were made on the limbus with a marker pen under slit-lamp examination in all patients before the surgery. The CALLISTO eye intraoperative navigation system (Carl Zeiss Meditec AG, Jena, Germany) was used to perform the surgeries. A 2.2-mm transparent corneal incision at 130° and a 5.4-mm central continuous circular capsulorhexis were made under the direction of the CALLISTO eye. After a standard divide-and-conquer phacoemulsification technique, a 909 M IOL was implanted into the capsular bag. The IOL axial alignment was adjusted to the implant axis, and incision watertightness was confirmed. The IOL power and axial alignment were calculated using IOL Master 700 and Barrett Toric formulas, with the surgical induced astigmatism (SIA) set to 0.3 D and the target refraction set to 0. Anterior CA and predicted posterior CA were taken into account while calculating the IOL power. Since CA would change from WTR to ATR with age [19], the postoperative residual astigmatism was set at -0.50 D due to the under-correction rule in patients of young age or with-the-rule CA.

### Postoperative follow-up and assessments

The patients were followed up at 1 week, 1 month, 3 months, and 1 year postoperatively. UDVA, BCVA, uncorrected intermediate visual acuity (UIVA), UNVA and subjective refraction were measured at every follow-up, while defocus curves and aberrations were measured at 3-month postoperatively. A detailed slit-lamp observation of the IOL axial alignment was recorded after the axis label of IOL was completely exposed under pupil dilation at the1-week, 1-month and 3-month visit. The functional vision was assessed using the modified Vision Acuity and Visual Function Index 14 (VF-14) [20] at 3 months. The patients also completed questionnaires regarding vision satisfaction, photic phenomena, and the presence of any vision disorder in daily life [21]. In addition, we recorded all the side effects or complications that occurred during the 1-year period.

### Statistical analysis

Quantitative variables were expressed as mean and standard deviation (mean ± SD). Qualitative variables were analyzed by absolute number (n) and frequency (%). *t*-tests were used to assess the between-group differences for continuous data, while χ^2^ tests were used to compare the categorical data. Comparisons between the three groups were performed using ANOVA and related-samples Friedman 2-way analysis of variance by rank. The relationships between the continuous variables were assessed using Pearson correlation analysis. Sample sizes of 17 for each group achieved 80.70% power to reject the null hypothesis of zero effect size when the population effect size is 1.00, and the significance level (alpha) was 0.050 using a two-sided two-sample equal-variance *t*-test. All statistical analyses were performed using SPSS Statistics version 26.0 (IBM/ SPSS, Inc., Chicago, IL). Statistical significance was set at *P* < 0.05.

## Results

### Baseline characteristics

**Fig. 1:**
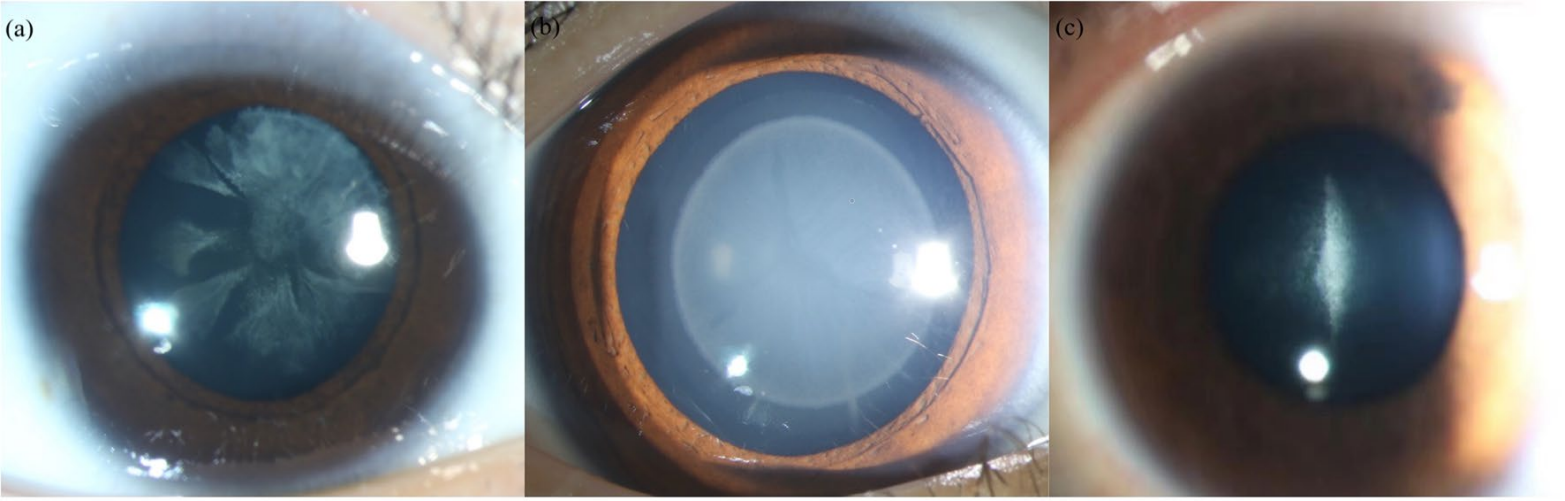
Classification of the three groups according to the anatomic position of the lens opacity. (**a**) Cortical group (**b**) Nuclear group (**c**) posterior subcapsular group

Fifty-nine eyes of 41 patients (17 male and 24 female) were enrolled in this study. Three patients (three eyes) were lost to follow-up, and one patient was excluded due to posterior capsular rupture during the surgery. Finally 55 eyes (37 patients) were followed up at least for 1 year; the mean follow-up duration was 386 ± 20 days. Nineteen patients (19 eyes) were unilateral cases, and 18 patients (36 eyes) were bilateral cases. Preoperative UDVA was 0.93 ± 0.41 logMAR, and preoperative CA ranged from 0.75 to 4.63 D. According to the anatomic location, the patients were divided into the cortical (11 patients/ 17 eyes), nuclear (12 patients/ 20 eyes), and PSC groups (14 patients/ 18 eyes) (Fig. 1). The baseline findings of the three groups are listed in Table 1. There were no statistically significant differences in baseline indicators except lens thickness. The predicted spherical equivalent (SE) was -0.19 ± 0.16 D. The predicted residual astigmatism was -0.33 ± 0.15 D.

**Table 1:** The baseline characteristics of patients in the study

Characteristics, mean ± SD	All (*n* = 55)	Cortical (*n* = 17)	Nuclear (*n* = 20)	Posterior subcapsular (*n* = 18)	P_C&N&P_
Age, y	26.7 ± 2.7	26.9 ± 1.8	26.3 ± 3.2	26.9 ± 2.8	.81
Axial length, mm	24.77 ± 1.66	24.59 ± 1.70	25.47 ± 1.89	24.19 ± 1.14	.08
Anterior chamber depth, mm	3.64 ± 0.36	3.82 ± 0.17	3.53 ± 0.40	3.65 ± 0.38	.13
Lens thickness, mm	3.62 ± 0.39	3.42 ± 0.23	3.80 ± 0.38	3.58 ± 0.43	.04
White-to-white, mm	11.84 ± 0.39	11.86 ± 0.41	11.83 ± 0.47	11.84 ± 0.29	.98
Flat keratometry, D	43.44 ± 1.28	43.64 ± 1.45	43.00 ± 1.31	43.73 ± 0.99	.16
Steep keratometry, D	45.50 ± 1.58	45.74 ± 1.57	44.93 ± 1.71	45.90 ± 1.30	.11
Corneal astigmatism, D	2.06 ± 0.79	2.10 ± 0.73	1.93 ± 0.68	2.17 ± 0.98	.34

*
P*_*C&N&P:*_* P* value of cortical, nuclear, and posterior subcapsular groups; *SD:* standard deviation

### Visual outcomes and defocus curves

**Fig. 2:**
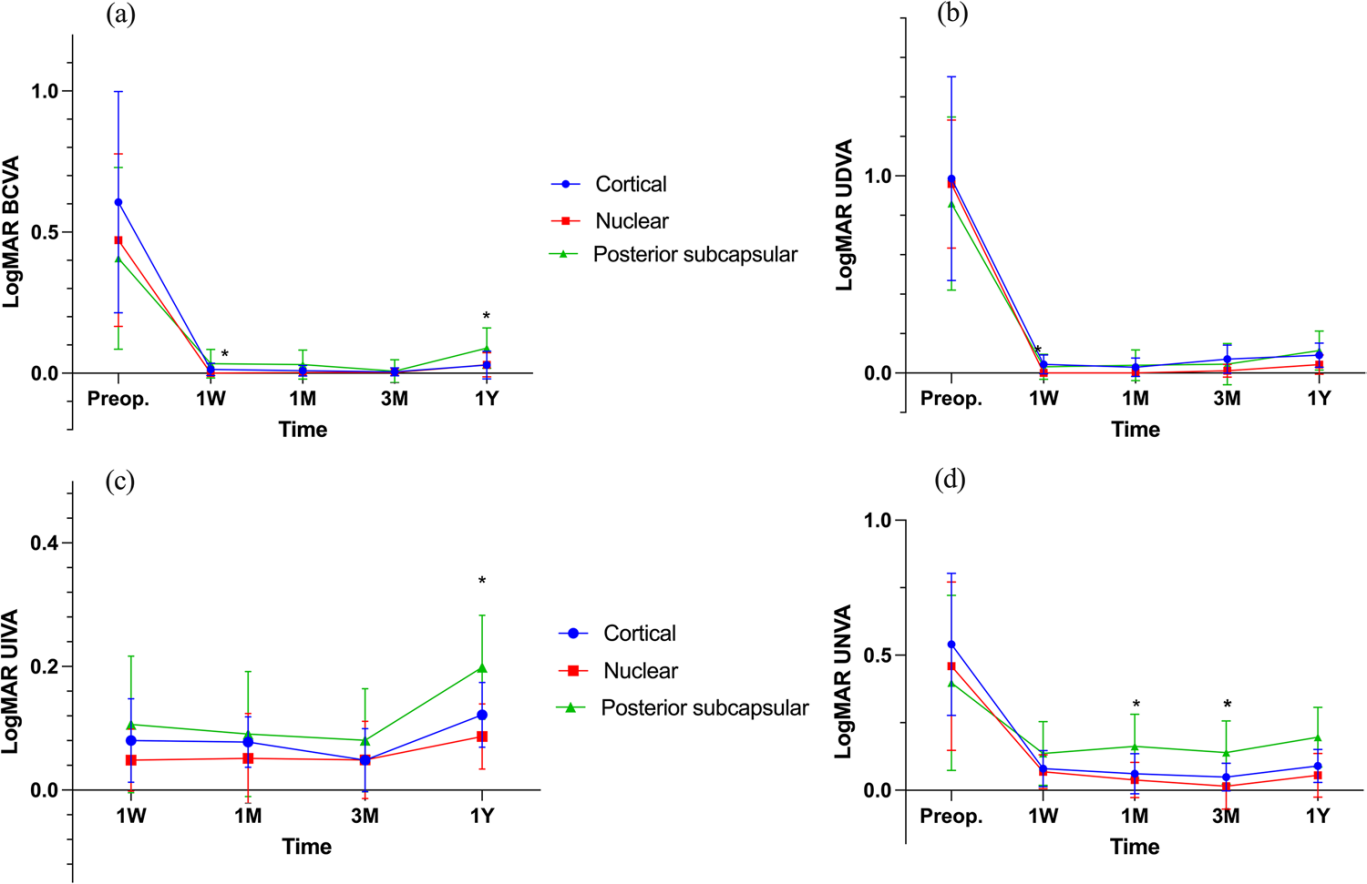
The preoperative and postoperative visual outcomes of the three groups at 1 week, 1 month, 3 months, and 1 year. All data are presented as a mean ± SD. (**a**) BCVA (logMAR) (**b**) UDVA (logMAR) (**c**) UIVA (logMAR) (**d**) UNVA (logMAR). *p < 0.05

The postoperative UDVA, UNVA, and BCVA were significantly better than those before the surgery in all groups. The postoperative SEs measured by automatic optometry were -0.21 ± 031 D (cortical group), -0.06 ± 034 D (nuclear group), and -0.10 ± 0.32 D (PSC group). Figure 2 shows no significant differences in BCVA, UDVA, and UIVA among the three groups at 1 week, 1 month, and 3 months postoperatively. However, compared with that of the PSC group, the nuclear group displayed better 1- and 3-month UNVA (1 month: 0.04 ± 0.07 vs. 0.16 ± 0.12, *p* = 0.007; 3 months: 0.01 ± 0.09 vs. 0.14 ± 0.12, *p* = 0.010) and 1-year BCVA (0.03 ± 0.04 vs. 0.09 ± 0.07, *p* = 0.044). Moreover, at the 1-year postoperative follow-up, UNVA and UIVA were significantly lower in the PSC group than that in the other two groups.

**Fig. 3:**
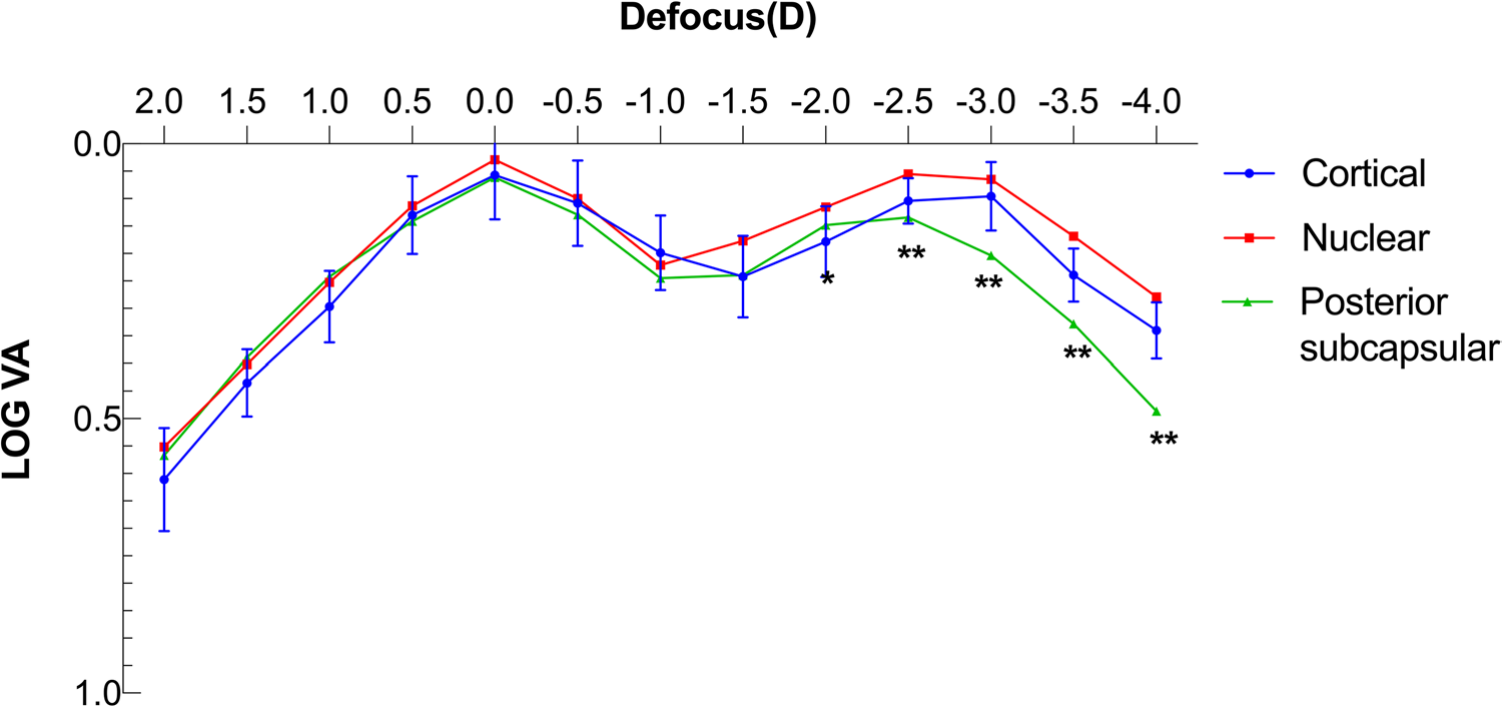
Monocular defocus curves of the cortical, nuclear, and posterior subcapsular groups at 3-month visit. All data are presented as a mean ± SD. *Significant difference (*p* < 0.05)

The postoperative defocus curves of all three groups are shown in Fig. 3. All defocus curves showed a bimodal pattern, with the far focus at 5 m and near focus at 40 cm (nuclear and PSC groups) or 33 cm (cortical group). All three groups displayed a continuous vision above 0.2 logMAR (20/40) between -0.50 to + 0.50 D and -2.0 to -3.0 D. The nuclear group had a wider depth of field, also providing VA above 0.2 logMAR (20/40) at -1.5 D and -3.5 D. Meanwhile, the defocus curve of the PSC group showed a worse near vision. Significant differences were detected between the nuclear and PSC groups from defocus -2.5 D to -4.0 D and between the cortical and PSC groups from defocus -3.0 D to -4.0 D (all *p* < 0.05).

### Residual astigmatism and postoperative IOL Stability

**Fig. 4:**
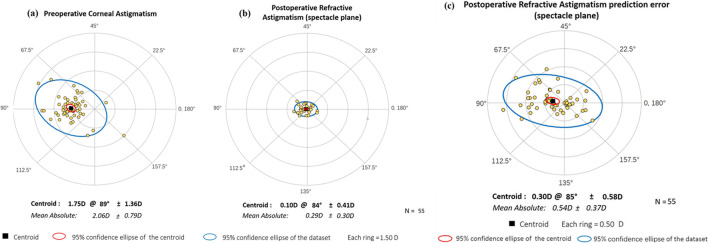
Double-angle plots of preoperative and 3-month postoperative astigmatism of all the patients. Each ring of (**a**) and (**b**) = 1.50 D. Each ring of (**c**) = 0.50 D

The residual and preoperative astigmatism are shown in the double-angle plots (Fig. 4). The preoperative mean CA was 2.06 ± 0.79 D, while the postoperative 3-month mean RA was 0.29 ± 0.30 D. Additionally, 85% and 100% of patients had a 1-year postoperative RA of < 0.5 D and < 1.0 D, respectively. The 3-month postoperative rotation was 2.48° ± 1.89°, with 90% of patients within 5° and no deviation of more than 10°. A IOL rotation of 9° was found in one case at 1-week follow-up, but no significant re-rotation was discovered in three visits within three months after IOL repositioning surgery (Supplemental Video 1), so the 3-month result of this patient was also included in the statistics. The postoperative RA prediction error was 0.54 ± 0.37 D.

### High-order aberrations and objective visual quality

At 3 months postoperatively, the HOA values were significantly better than those preoperatively in all groups (0.20 ± 0.15 vs. 0.62 ± 0.39, *p* < 0.001). Similar results were observed for objective visual quality, such as SR (0.02 ± 0.03 vs. 0.16 ± 0.14, *p* < 0.001) and the MTF curve (MTF-10, 0.08 ± 0.05 vs. 0.41 ± 0.18, *p* < 0.001; MTF-30, 0.02 ± 0.03 vs. 0.13 ± 0.08, *p* < 0.001). Table 2 shows that the total HOAs, MTF-10, and MTF-30 of the PSC group were significantly different from that of the other two groups. The subgroups of HOAs, such as trefoil, were also significantly different between the PSC group and the other two groups. Further, the PSC group showed a significantly lower SR (0.09 ± 0.04 vs. 0.21 ± 0.17, *p* = 0.031) than the nuclear group at 3 months postoperatively.

**Table 2:** High-order aberrations and objective visual quality evaluation index of three groups at 3 month after surgery in 55 eyes of 37 patients

Aberrations, Mean ± SD	All (*n* = 55)	Cortical (*n* = 17)	Nuclear (*n* = 20)	Posterior subcapsular (*n* = 18)	P_C&N_	P_N&P_	P_C&P_
Preoperative HOAs total [μ]	0.62 ± 0.39	0.74 ± 0.36	0.57 ± 0.48	0.60 ± 0.23	.484	.882	.618
Preoperative MTF-10 total	0.08 ± 0.05	0.05 ± 0.03	0.09 ± 0.06	0.07 ± 0.05	.228	.430	.663
Preoperative MTF-30 total	0.03 ± 0.02	0.02 ± 0.02	0.03 ± 0.02	0.03 ± 0.03	.515	.840	.461
Preoperative SR total	0.02 ± 0.03	0.02 ± 0.02	0.03 ± 0.03	0.02 ± 0.01	.572	.450	.903
Postoperative total							
HOAs [μ]	0.20 ± 0.15	0.15 ± 0.08	0.16 ± 0.11	0.29 ± 0.19	.857	.023	.031
Coma [μ]	0.09 ± 0.07	0.08 ± 0.05	0.07 ± 0.04	0.13 ± 0.10	.681	.028	.113
Spherical [μ]	0.03 ± 0.02	0.03 ± 0.02	0.03 ± 0.03	0.02 ± 0.02	.534	.856	.461
Trefoil [μ]	0.12 ± 0.09	0.08 ± 0.07	0.09 ± 0.06	0.18 ± 0.10	.831	.008	.012
Secondary astigmatism [μ]	0.03 ± 0.02	0.03 ± 0.01	0.02 ± 0.01	0.04 ± 0.02	.229	.062	.589
MTF-10	0.41 ± 0.18	0.46 ± 0.17	0.49 ± 0.17	0.28 ± 0.12	.630	.003	.021
MTF-30	0.13 ± 0.08	0.15 ± 0.08	0.15 ± 0.09	0.08 ± 0.03	.928	.031	.048
SR	0.16 ± 0.14	0.15 ± 0.12	0.21 ± 0.17	0.09 ± 0.04	.351	.031	.286

*HOAs* high-order aberrations, *MTF* modulation transfer function, *P*_*C&N*_ *P* value of cortical group and nuclear group, *P*_*N&P*_ *P* value of nuclear group and posterior subcapsular group, *P*_*C&P*_ *P* value of cortical group and posterior subcapsular group, *SD* standard deviation, *SR* Strahl ratio

### Quality of life and postoperative complications

**Fig. 5:**
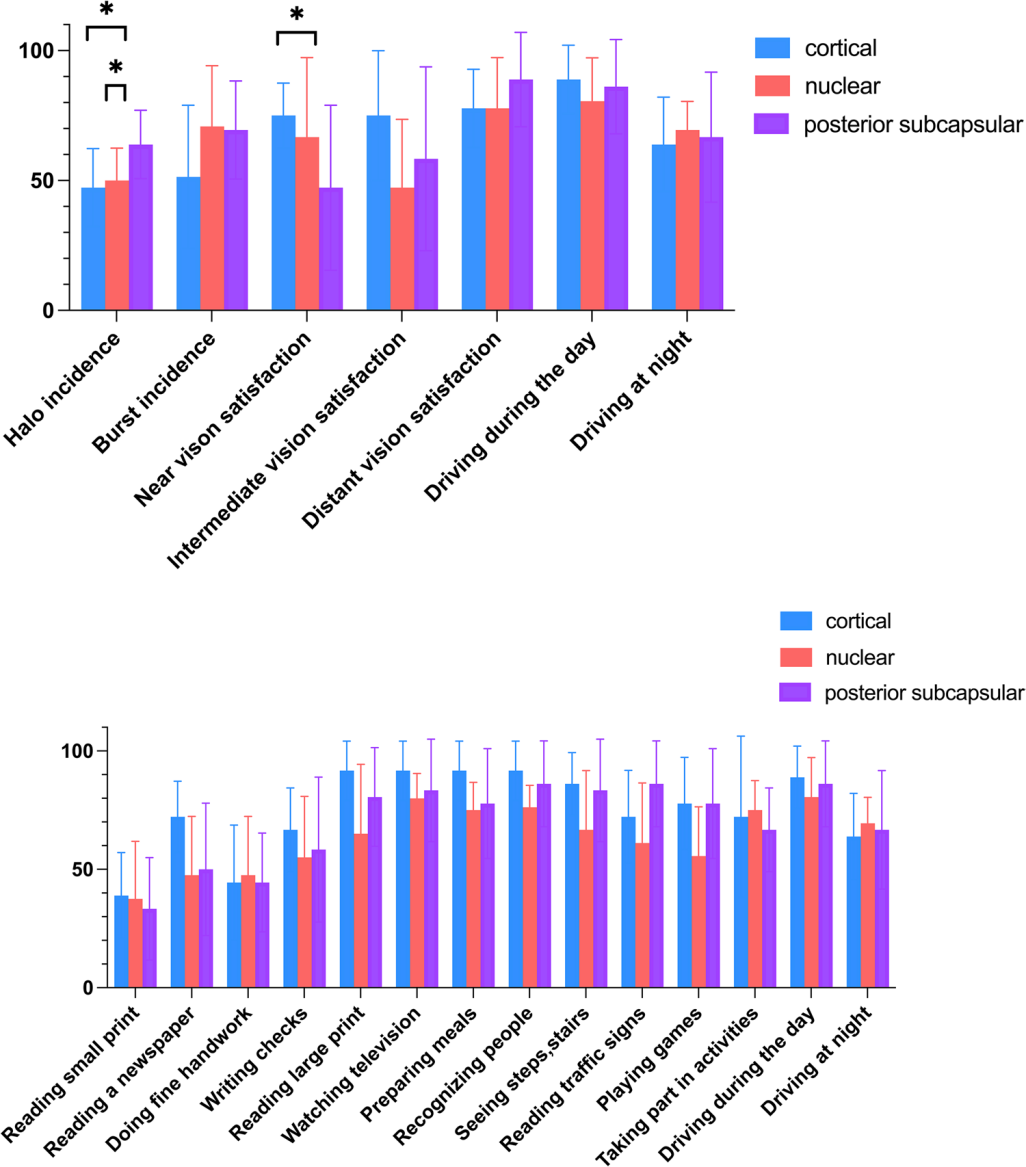
Subjective visual quality questionnaire classification bar chart of the three groups at 3 month. Top: Vision satisfaction and photic phenomenon questionnaire. Bottom: VF-14 questionnaire. Y axis: The incidence of the optic phenomena and the score of the visual satisfaction. *Significant difference (*p* < 0.05)

The VF-14 questionnaire was completed by each patient. The mean score was 69.58 ± 25.37, and there were no significant differences among the groups (*P* > 0.05). Figure 5 shows that among all measures of vision satisfaction, driving satisfaction was the highest (85.19%), while night vision satisfaction was relatively low (66.67%). Distant vision satisfaction was quite high (81.48%), while intermediate (60.19%) and near vision satisfaction (62.96%) were relatively low. Among the photic phenomena, the incidence of halo (53.70%) and starburst (63.43%) were relatively high. The near vision satisfaction was lowest in the PSC group (47.22%) among the three groups (*p* = 0.04, *p* = 0.02). Similarly, the PSC group had a significantly higher incidence of halo than that in the other two groups. Furthermore, the incidence of halo was significantly higher in patients who underwent unilateral surgery patients (63.46%) than in those who underwent bilateral surgery (44.64%) (*p* < 0.001). One year after surgery, 8 patients (14.5% in all, 11.8% in cortical group, 10.0% in nuclear group, and 22.2% in PSC group) with recurring VA decrease were diagnosed with posterior capsule opacification (PCO), and underwent YAG laser posterior capsulotomy to restore their vision.

## Discussion

To our knowledge, this study is the first report that analyzes and compares the visual quality, feasibility, and stability of TMIOLs in adult patients with different types of DC. All postoperative visual outcomes showed a significant improvement in all groups. With the excepted correction of preoperative refractive error, the far, intermediate, and near VA of all the patients were significantly improved. The reason for the lower postoperative VA at 1 year than at the early stage was probably due to the incidence of PCO, which was as high as 14.5% at 1 year follow-up because of the hydrophilicity of IOLs and the younger age of this population of patients [22]. Although the IOLs have a design of hydrophobic surface to avoid PCO, the incidence of such complication in our study can be considered as high.

In terms of IOL rotational stability, although one IOL repositioning surgery was performed, the mean rotation measured at the 3-month follow-up was 2.48 ± 1.89°, with 90% of patients showing a rotation < 5°. Our findings confirm that the implantation of plate-haptic 909 M IOLs provides a safe and stable astigmatism correction in adult congenital cataract patients with CA. Although the diameter of the 909 M IOLs is only 11 mm, the four-haptic design provides strong support from the four corners of the IOLs, and absence of gap between the haptic and the optic, increasing stability of the IOLs in the capsular bag. Moreover, the findings of our previous study discovered that the main factors affecting IOLs rotation were lens thickness and axial length. Because of the thinner lens thickness and < 26 mm axial length, the rotation stability of IOLs in young patients was relatively higher than that in elderly patients. Furthermore, in order to reduce early exercise-induced rotation, all the patients were hospitalized and observed continuously for 3 days after surgery [23].

At 3 months postoperatively, the defocus curves showed that cortical and nuclear groups had significantly better vision than the PSC group at near focus, indicating that the PSC group had an unsatisfactory near vision. Objective visual quality quantified by HOAs, SR, and the MTF curve [24–26] were significantly improved postoperatively in all groups, while between-group comparisons showed that the cortical and nuclear groups had significantly better MTF-10 and MTF-30 values than the PSC group. This further confirmed that the patients in the PSC group had slightly poorer near vision and lower visual quality. Similarly, the functional vision evaluated by the VF-14 scores showed a satisfactory result in all groups despite the limitations in fine object recognition, especially in the PSC group. We presumed the probable reason is that the light energy is divided into near (35%) and far focus (65%) in MIOLs, which reduces the contrast of the retina and leads to a decrease in contrast sensitivity, especially in high spatial frequencies [27–29], thus causing difficulties in reading small print. Therefore, choosing a MIOL for patients with posterior subcapsular opacity who require excellent near vision must be considered carefully since the postoperative near vision may not meet their requirements completely.

Similar results were observed for HOAs, which are responsible for postoperative visual complaints such as halo, starburst, and blurred vision. At 3 months postoperatively, HOAs of the total eye were higher in the PSC group than that in the other groups. This suggested that the PSC group may report more incidences of visual disorders after surgery, which was supported by the survey about photic phenomena. The responses to the photic phenomenon questionnaire also proved that the incidence of halo was much higher in the PSC group than that in the other two groups. Meanwhile, the incidence of halo was slightly higher in unilateral surgery cases (63.46%) than that in bilateral surgery cases (44.64%). The reasons for this finding were as follows: (1) Diffractive MIOLs formed two simultaneous foci with different light dispersion; the visual cortex then suppressed the fuzzy focus and strengthened the clear one [30], Given the poorer vision of the PSC group, it was difficult to obtain a clear focus, resulting in blurred vision and enhanced halo; (2) The high proportion of patients who underwent unilateral surgery in the PSC group (55.56%, 10/18) led to a high incidence of halo, while the proportion was 29.41% (5/17) in the cortical group and 20.00% (4/20) in the nuclear group. Due to their excellent contralateral eyesight, the blurred images of the operated eyes were suppressed, resulting in a more “monocular” rather than stereoscopic vision and decreased fine object resolution [31]. Meanwhile, in the process of binocular stereoscopic vision reconstruction, the discomfort caused by binocular competition was more troubling than the loss of stereoscopic vision [32]. However, the high driving satisfaction findings also indicated that photic phenomena were not severe enough to hamper night driving [33].

The limitations of this study were firstly due to the relatively low DC prevalence, the sample size was not large enough, resulting in a relatively small number of cases in each group, and should be expanded in future large-scale, multi-center studies; Secondly, photic phenomena were only measured using questionnaires rather than objective instruments, which makes this a semi-quantitative rather than a quantitative finding.

## Conclusion

Our study provides novel data about the feasibility and effectiveness of TMIOLs implantation in adult patients with DC accompanied by CA. At Lisa 909 M IOLs implantation in these patients achieved good postoperative visual outcomes and significantly reduced glasses dependency. DC patients with cortical or nuclear opacity showed better whole-course VA and quality of vision. However, TMIOL implantation in DC patients with posterior subcapsular opacity should be made an emphasis of the possibility of postoperative unsatisfactory near vision and photic phenomena. Detailed preoperative communication is essential.

### Supplementary Information

Below is the link to the electronic supplementary material.

Supplementary file1: Video 1: The IOL repositioning surgery of a patient with a IOL rotation of 9°. 20 days after surgery, the patient was conscious of blurred vision and went to the outpatient department for reexamination. The intraocular lens was found to be located at 69° (implant axis 78°), and the refraction was +0.50DS-1.00DC*135°→1.0 (Snellen 20/20). (MP4 76951 KB)

Supplementary file2 (DOCX 16 KB)
