# Supplementary material for: Comparison of visual outcomes in adult patients with different types of developmental cataracts after toric multifocal intraocular lenses implantation
Source: Graefes Arch Clin Exp Ophthalmol. 2023 Jun 29;261(12):3521–30. doi: 10.1007/s00417-023-06148-4 (PMC10667390; doi:10.1007/s00417-023-06148-4)
Supplement: Supplementary file 2 — Supplementary file2 (DOCX 16 KB) [file 417_2023_6148_MOESM2_ESM.docx]

**Optical Phenomena Questionnaire**

1. Do you find that your sight at present in some way causes you difficulty in daytime driving?（0=Very great difficulty, 1=Great difficulty，2=Some difficulty，3=A little difficulty，4=No difficulty)
2. Do you find that your sight at present in some way causes you difficulty in night driving?（0=Very great difficulty, 1=Great difficulty，2=Some difficulty，3=A little difficulty，4=No difficulty)
3. How satisfied are you with your distant vision?(0=Very dissatisfied 1=Generally acceptable 2=Satisfied 3=Quited satisfied 4=Very satisfied)
4. How satisfied are you with your intermediate vision?(0=Very dissatisfied 1=Generally acceptable 2=Satisfied 3=Quited satisfied 4=Very satisfied)
5. How satisfied are you with your near vision?(0=Very dissatisfied 1=Generally acceptable 2=Satisfied 3=Quited satisfied 4=Very satisfied)
6. How often do you experience haloes?(0=Never, 1=Rarely，2=Sometimes，3=Often，4=Always)
7. How often do you experience starburst?(0=Never, 1=Rarely，2=Sometimes，3=Often，4=Always)
